# The Hospital Nursing Supplement

**Published:** 1896-01-18

**Authors:** 


*The Hospital*, January 18, 1896. *Extra Supplement.*

### "The Hospital" Nursing Mirror.

Being the Extra Nursing Supplement of "The Hospital" Newspaper.

[Contributions for this Supplement should be addressed to the Editor, The Hospital, 428, Strand, London, W.C., and should have the word "Nursing" plainly written in left-hand top corner of the envelope.]

### News from the Nursing World.

### NURSES AT WINDSOR.

An excellent performance of Handel's oratorio of "The Messiah" took place at the Leopold Institute, Slough, last week, in aid of Princess Christian's Trained Nurses' Fund. The concert, under the patronage of the Queen, was given by the Windsor and Eton Choral Society, and Her Royal Highness Princess Christian herself took part in the performance. Princess Victoria of Schleswig-Holstein was present amongst the audience, which was an attentive and appreciative one. The Nurses' Fund should benefit substantially by the efforts made for it.

### REGISTRATION OF NURSES.

The discussion at the meeting called by the Parliamentary Bills Committee of the British Medical Association, on Tuesday, resulted in the passing of a resolution, by six votes to five, against State registration for nurses. The unrepresentative character of the conference may be gathered from the circumstance that not a single nurse-training school was represented except Middlesex, which voted against State registration. It is further interesting to record those who voted for State registration, viz.: Dr. Bedford Fenwick, Mrs. Bedford Fenwick, Miss Isla Stewart, Miss Annesley Keneally, and another; against State registration Dr. Fardon (Middlesex Hospital), Miss Wedgwood (Royal British Nurses' Association), Miss Wilson (Workhouse Nursing Association), Mr. Burdett (Pension Fund), the representative of the Glasgow Maternity, and another. The members of the Parliamentary Bills Committee, or some of them, expressed honest indignation at Dr. Bedford Fenwick's announcement that a Bill for the State registration of nurses would be introduced forthwith, independently of the proceedings at the British Medical Association and the conference. Mrs. Fenwick having remarked that more nurses voted with her than against her in the division, it was pointed out to her that she had with her two of the same household—in fact a family party, which caused the conference to end in a pleasant ripple of laughter.

### A VACANCY AT EAST GRINSTEAD.

The gradual awakening of guardians to the consciousness of the new responsibilities which the employment of trained nurses necessarily entails upon them, is not without interest to observers of the process. The path of those who have no previous knowledge of what constitutes the "training" of a nurse is naturally beset with stumbling blocks. At East Grinstead, for instance, the Board appointed a nurse recently of whom a guardian remarked (according to the local press), "I was very pleased with her appearance and manner, and I considered her fully competent." However, after about a fortnight's residence in the infirmary the nurse resigned, saying that she understood from the matron and doctor that she would have to give lectures to probationers, which she apparently felt incompetent to undertake. It seems extraordinary that details of her duties were not explained to her before she was engaged, as systematic training of probationers can only be efficiently done by a nurse with special knowledge and experience. The guardians would have avoided a blunder, and the present nurse would have escaped disappointment had the Board seen fit to invite the assistance of the medical officer and the matron in their selection of a candidate for so important a post. As the matter has now been referred to the Visiting Committee, it is possible that they will take pains to have the duties and position of the next nurse clearly defined before her appointment.

### DISTRICT WORK AT PLAISTOW.

The summary of cases attended last year by the nurses from the Plaistow Maternity and District Nurses' Home has been made out, and shows a total of 3,164 patients, to whom 86,436 visits were paid; 1,299 persons attended at the Home for dressings, massage, &c.; whilst the loans of linen and other nursing requisites made by Sister Catherine's staff of nurses during last year numbered 1,299.

### OUR NEEDLEWORK COMPETITION.

"A thousand thanks for the parcel of nice things so kindly sent for my poor friends here," writes the sister-in-charge of Gort Hospital; and warm thanks have also come from the matrons of all the institutions to which we had the pleasure of taking or sending the delightful clothing provided by our readers for hospital patients. There is no doubt of the practical value of such useful garments to men and women about to exchange hospital comfort for the poor surroundings of their homes at a trying season of the year. Will all our friends kindly bear in mind that there is an urgent and constant demand for flannel shirts for men?

### CORRESPONDENCE CLASSES.

There are many advantages to busy people in a scheme which provides for them a course of systematic study without interfering with their other duties, and these have doubtless influenced a number of district nurses to join Miss Lamport's Correspondence Classes. It is convenient to be able to send in the papers when leisure permits, instead of having to comply with a hard-and-fast rule as to date. But the classes are popular with others besides nurses, many ladies in the country being glad of help in the study of physiology and hygiene. Pupils who wish to work for a certificate are prepared for the examination of a well-known society, and all those who have followed this course have passed successfully.

### FORMER PATIENTS ENTERTAINED.

For the fifth time the nurses at the Middlesbrough Home have assisted at a special annual party, which passed off as brilliantly as its predecessors, thanks to the able administration of the matron, Miss Purvis. The guests, some five hundred in number, were all children, many of them former patients, and they did full justice to an excellent tea. An entertainment was afterwards given, and concluded with an exhibition of living waxworks, which was highly appreciated. A magnificent Christmas tree was provided by Lady Zetland, and many other friends sent toys for distribution. The kind efforts of Miss Purvis and her nurses to give a merry Christmas to old patients receives much local encouragement.

### FIRE AT MULLINGAR ASYLUM.

No loss of life occurred at the fire which broke out last week at the Mullingar Lunatic Asylum, although some parts of the building were destroyed. Owing, it is said, to the promptitude and energy displayed by the whole staff of officials, all fatalities were avoided.

### WARWICK DRISTICT NURSING.

At a recent meeting held in the Court House at Warwick, a lecture on Mexico was given by the Earl of Warwick, and the Countess of Warwick made an appeal on behalf of the District Nursing Association. In the course of a charming little speech the obvious advantages of trained nursing for the sick poor were touched on, and also the urgent need for increased subscriptions. Lady Warwick suggested that the town should be canvassed for the latter, and remarked that two nurses were much wanted, and she hoped they would eventually be secured. During the year 117 patients have been attended by Nurse Moore, and she has paid them 3,050 visits. The present subscriptions do not cover one nurse's salary, and therefore need to be substantially augmented to meet future expenses.

### WOMEN ON HOSPITAL BOARDS.

The suggestion to nominate a woman as one of the managers on the board of Edinburgh Royal Infirmary has naturally led to considerable discussion. It is plain that a lady might fill such a post with advantage to any institution, if duly equipped for the work. A candidate for the vacancy at Edinburgh has been already mentioned, but it is not stated if she has had any previous acquaintance with hospital administration. A contemporary points out the valuable advice which a lady might give the male managers in such feminine departments as the laundry, kitchen, or the nursing; but the worth of her counsels must depend on her practical knowledge of these branches, and the contributors to the Infirmary, in whose hands the election lies, are hardly likely to nominate any lady who lacks it. It is therefore fair to imagine that the candidate for whom the innovation is contemplated is possessed of wide experience of patients, nursing, and general management. It may be urged that men are elected to similar appointments without preliminary training, but as the addition of women to boards and committees is expressly done on account of their supposed usefulness, it is essential for the credit of the sex that the first selection should be a wise one.

### THE WOUNDED IN AFRICA.

It is with great satisfaction that all who have friends in South Africa hear good reports of the arrangements made for the wounded after the recent engagement with the Boers. The telegram of a special correspondent calls attention to the devotion of the nurses, and the care and comfort provided for the sufferers, and the admirable hospital accommodation which was promptly secured to them.

### PROVISION IN SICKNESS.

A correspondent writing in *The Trained Nurse* calls attention to the extremely sad case of Nurse Eliza Cook, who went over to America about three years ago. On the authority of a Pittsburg paper, it is stated that she was a patient at the Allegheny General Hospital, but eventually became so violently mad that she could not be kept there, and was transferred to the insane department of the Poor Farm. It appears that this means, according to this correspondent, association with "the most neglected and unfortunate of all human creatures." This painful picture is brought forward as an incentive to press forward the formation of a national association of nurses for mutual benefit in sickness and health. The terms in which the arrangements for lunatics are spoken of seem to show that reform is urgently needed there.

### OUR SISTER IN INDIA.

On New Year's Day Sister Evelyn Mary Leicester, of the Indian Nursing Service, died of enteric fever at Allahabad. She had been working at the Station Hospital, where the cases of enteric had been unusually numerous, and probably contracted the disease in the course of her duty. The sad news of Miss Leicester's death reached her sister in London by telegram, and a cheerful letter since received from Allahabad, dated December 19th, shows that the illness which ended fatally was only a short one. Miss Leicester was trained at the Radcliffe Infirmary, Oxford, Queen's Hospital, Birmingham, the Central London Sick Asylum, and Clapham Maternity Hospital. In 1893 she was appointed to the Indian Nursing Service, and went out in November of that year, enjoying fair health during her two years' work in India, a slight attack of malarial fever being the only illness recorded during that time. Miss Leicester was only twenty-eight, and her unexpected death has caused great shock and grief to many friends in the nursing world by whom she was loved and valued.

### PROGRESS IN THE COLONIES.

News comes to us from Australia that Miss Catherine H. Spence forms one of the Royal Commission appointed to investigate the state of affairs at the hospital at Adelaide. The occasion is memorable in the colony as being the first on which a woman in Australia has had a seat on a commission.

### SHORT ITEMS.

An account of the Medical Mission at Florence draws attention in a gratifying manner to the able assistance which the medical officer receives from lady dressers, but it is not explained if these are students who go through the complete education necessary to qualify for women doctors.—Miss R. C. Despard, M.B.London, has been appointed a junior assistant medical officer at the Holloway Sanatorium, Virginia Water.—On the recommendation of the Medical Superintendent and the Matron, the Board of Guardians of the Lambeth Infirmary have decided to adopt the three years' system of training for probationers.

### Lectures on Nursing.

By a Superintendent of Nurses.

### V.—THE ADMINISTRATION OF MEDICINE.

The giving of medicine is a very important matter, and there are one or two rules that should be invariably followed, as any carelessness in the use of drugs may end in some sad disaster, embittering a nurse's whole life. (1) Never give any medicine from a bottle without a label. (2) Never give a dose without reading the label, as the strength may have been altered, or the quantity of the dose changed, without your having noticed any alteration of the prescription. (3) Never give medicine if you have the slightest doubt about its being right, as mistakes may occur even in a dispensary. I have known a nurse give a patient lotion for medicine, and apply the latter to his leg. I remember another nurse who, many years ago, administered a night draught to a man without noticing the direction, and emptied a small bottle containing four times the quantity of chloral. It was well for her that he had been a great drinker; had this not been the case the man would probably never have awakened from the sleep thus induced. Do not trifle with matters of this kind; always keep lotions in a separate place from medicines, see that the bottles containing them are of blue glass, labelled "Poison," and be sure to put them out of reach of your patients in a locked cupboard.

In one hospital I was in, many years ago, a man of a highly nervous temperament sprang out of bed, seized a bottle of opium on a table near him, and drank the contents; fortunately the bottle had only a little in, but sufficient to make it necessary to keep the patient walking about the whole night, and by rough means to prevent him from falling asleep. If you have omitted giving a dose at the right hour, do not make up for this by giving a double dose next time. You do not always know the potency of the drugs contained in the medicine. I remember an instance of a doctor, who being ill and under treatment, and having had his medicine at the right time, insisted on his attendant giving him another dose long before it was due, saying he knew quite well the quantity of chloral he could take. The unfortunate attendant listened to his patient, and disobeyed orders, the consequence being the almost instantaneous death of the sick man.

In pouring physic out, hold the bottle so that the label is uppermost, and thus avoid soiling it. If a patient is delirious (or a child obstinate) and refuses to take medicine, gently but firmly hold the nose; he will then be obliged to open his mouth to enable him to breathe, when you can pass a spoon far back in the mouth, and empty it slowly. Do not give patients any medicine not ordered or sanctioned by the doctor—indeed, none should be given which is not ordered in writing; and here let me say how wrong it is for nurses to doctor themselves or each other. I have known cases in which they have caused serious alarm by infringing this rule.

### Prescriptions.

It is well you should be able to read prescriptions, and you should do this constantly, so as to become familiar with the words and signs usually employed.

A model prescription consists of four parts: the Superscription, the letter ℞, a sign which is an abbreviation of the imperative mood of the verb "Recipio"—it is also an astrological symbol representing Jupiter, whose ascendancy favoured the collection of herbs; the Inscription, the names and doses of drugs prescribed; the Subscription, directions to dispenser; the Signature, instructions for patient.

The ingredients of a typical prescription are representative of the following: (*a*) the basis or active ingredient; (*b*) the adjuvant or auxiliary; (*c*) the corrective; (*d*) the vehicle.

For example: ℞. (*a*) Magnes. Sulphatis., ʒii.; (*b*) Magnes Carbonatis., gr. xx.; (*c*) Syrup. Zingiberis, ♏xx; (*d*) Aq. Menth. Pip. ad, ℥i.; Sig. Cap. ℥i. ter in die.

There are a good many classes of drugs; some of them no doubt you are quite familiar with. Emetics, to induce vomiting, for instance, mustard and water; purgatives, which increase the secretions of intestines and cause them to expel their contents, such as magnesia; astringents, which lessen secretions, such as chalk; expectorants, which favour discharge of secretions from the respiratory organs, such as squills; stimulants, which increase the activity of the heart, as ammonia; anodynes, which relieve pain, such as opium; aromatics, which relieve spasms in the intestines, such as ginger; sedatives, which depress vital action, such as bromide; diuretics, which increase amount of urine, such as spirits of nitre; diaphoretics, which induce sweating, such as acetate of ammonia. It is very important a nurse should learn the effects of various drugs, that she may be able to tell if the medicine is producing undesirable symptoms, and to report accordingly, so that the doctor may discontinue their use; for instance, pot. iod.—potassium iodide—sometimes causes iodism, *i.e.*, bronchial catarrh, increased flow of mucus, coryza, increased flow of tears dripping from the nose; headache; and acne (or skin eruption); quinine and cinchona bark produce cinchonism, or buzzing in ears, deafness, and headache; antipyrin, scarlatina-like rash, cardiac depression, and faintness; nux vomica or strychnine, spasm of larynx and gastric disorder; salicylate of soda, depression, deafness, and delirium; hydrargyum (mercury, calomel) produces salivation, *i.e.*, profuse saliva, swelling of gums and mouth, foul breath, diarrhœa, and muscular tremors; arsenic, headache, soreness of eyes, muscular trembling, diarrhœa; opium, dry and foul tongue, contracted pupil, insensibility, and stertorous breathing, &c. Some medicines can be taken by patients better at one time than another. Cod liver oil is most easily taken the last thing at night, and, indeed, can be taken then in some instances when the patient cannot take it at any other time. It is always best to give it on a full stomach. Children can frequently be induced to take it in the form of cod-liver oil and maltine.

For a long time fruitless attempts were made to produce a tasteless castor oil, and at last Messrs. Allen and Hanbury have succeeded in their efforts and supply oil which is as free from taste as possible. Some people prefer taking it in coffee, but I think the most agreeable way is with lemon. The medicine glass should be rubbed with the lemon to the very brim inside, and a few drops should be dropped into the oil. The patient can then swallow it easily without the slightest nausea. To clean glasses which have had oil in, a little whiting rubbed round them will absorb the oil, after which they can easily be washed. If no whiting is at hand a little common plaster will act in the same way, taking up the grease.

Arsenic and iron should be taken after food, but quinine, in tonic doses, before a meal. If iron be given in the form of powder, it is best put on slices of bread and butter. If taken as a fluid it should be sucked through a tube or straw to avoid discolouring the teeth or injuring them. Drops should always be measured in a minim glass.

### Bed-sores.

One of the chief things a nurse should remember is her patient's liability to contract bed-sores. Such a large proportion of these being the result of carelessness, a nurse should, as a rule, feel it a great disgrace for any patient under her care to develop one. Of course, there are exceptions to this rule, and however much care is taken to prevent their forming, some cases will contract them, and sometimes even in one night. Œdematous patients are very liable to get them, and paralysed persons have to be carefully watched, because, feeling no local pain, they are not conscious of any discomfort. Bed-sores are caused by pressure and moisture. Helpless patients should be frequently moved, so that their position be changed, and the prolonged pressure on one spot be avoided. The principal parts to expect them are on those most prominent—sacrum, the great trochanters, shoulders, elbows, and heels. A very heavy or very thin person will be most liable to them. Always see that there are no rucks in the under-sheet, that it is also dry; make use of pads where necessary, and keep the patient as clean as possible, washing the exposed parts frequently during the day with soap and water; after washing and drying them thoroughly, apply the methylated spirits, and dust the part with zinc powder. Should the skin be actually abraded zinc ointment or a mixture of equal parts of Balsam of Peru and white of egg will be found a most comforting application. Amadou plaster is a favourite remedy with some doctors, a hole being cut in the centre as large as the sore, which may be dressed with one or other of the preparations referred to. If you ever find a patient has a sore, however small, be sure and tell the doctor at once — that is when you are the responsible nurse; but if you are a probationer, and notice any redness or any broken skin, immediately inform the sister, as delay in treatment may bring about a great deal of harm; occasionally one suffering from a long, troublesome complaint has conquered that, but has succumbed to bed-sore. At times nurses are afraid to speak of them, fearing they may be blamed, and hope to heal the sores before the surgeon finds out their existence. Now this is highly reprehensible; a bed-sore is often an index to a person's state of health, and it is most important to tell the medical man if there is one. I remember an instance in which a patient's temperature went up, and continued high, whilst there seemed nothing to account for it. The surgeon so thoroughly trusted the nurse that he thought that she would be sure to tell him if there was anything of that nature. Suddenly it appeared that the poor creature had a nasty sloughing sore on her back, but the nurse, who really was most devoted, hoped to get it well in a few days, and so had not mentioned it. Sometimes the skin may break; the subjacent tissues, however, remaining healthy, it soon heals. Another state of affairs may exist, in which you find the skin unbroken, though looking red; this is soon followed by blackness, and suddenly the skin gives way, revealing a sloughing, dirty wound, that may take months to heal.

Do not wait for the patient to tell you his back troubles him, but look constantly for yourself. A great danger to be anticipated from a bed-sore in the back of long standing is lest the discharge should burrow down to the spinal canal. Sometimes it is only necessary to attend to a patient's back once or twice a day, but in other cases, where there is incontinence of fæces or urine, constant care is required.

### Royal British Nurses' Association.

### A LIVELY MEETING.

The quarterly meeting of the Royal British Nurses' Association on the 10th inst. was a somewhat inharmonious one. Several of the nurse members who were present came away, resolved to absent themselves in future from gatherings where even their Royal President's influence has failed to secure the common courtesy and decorum which characterise most other official meetings.

The treasurer's report showed a balance of £122 10s. 6d. in the bank, hence the financial prospect is at present more promising than might have been imagined from the prognostications of bankruptcy with which a former treasurer is reported still to assail the Association.

Amongst other items the Executive Committee reported the result of a motion in the High Court of Chancery against the corporation, standing in the name of Barlow *v.* Thorne and others, in which a nurse had brought an action against the Association.

It was in connection with this case that a motion, put forward by Dr. Bedford Fenwick, gave rise to the heated discussion already alluded to. The motion appeared on the agenda of the General Council meeting as follows:—

To call attention to the recent successful proceedings taken by a registered nurse against the Executive Committee of the Corporation in the Chancery Division of the High Court of Justice, and to move a resolution.

The purport of the said resolution did not transpire, but the ruling of the chairman in the matter was opposed by Dr. Fenwick until Her Royal Highness authoritatively interposed, and a motion was carried that Dr. Bedford Fenwick be no longer heard. The circumstances attending the action taken by this nurse had appeared to Princess Christian of so much importance to the Association that she had convened a special general meeting to consider the case on January 28th, when the following resolution would be moved:—

That this meeting is of opinion that the conduct of Miss G. E. Barlow, a member of the Royal British Nurses' Association, in writing a letter, complaining of the management of the Association, to a public newspaper without previously communicating with the Executive Committee or bringing her grievance before the proper authorities, and in subsequently bringing an action at law against the Corporation and its officers, was disloyal and unjustifiable; and it further desires to record its strong disapproval of any member of the Association resorting to litigation with the Corporation, or inciting or encouraging a member to do so, until every possible means of settling the matter in dispute within the Corporation itself has been tried.

The correspondence between the registered nurse and the officials of the Association to which she belongs had been printed and sent round to members of the Council before the meeting. These letters, with an exhaustive reply addressed to Miss G. E. Barlow by the Executive Committee of January 3rd, are too long for insertion in our columns, and would probably possess but limited interest to the generality of readers.

### Royal London Ophthalmic Hospital, Moorfields.

The annual concert given by the matron, Miss Robinson, and the house surgeons of the Moorfields Ophthalmic Hospital on Thursday, January 9th, was an admirably-organised and very pleasant entertainment. A good programme had been arranged with the aid of many kind helpers, and was carried successfully through, and from the first item to the last was evidently appreciated by the audience. The concert was held in one of the out-patient rooms, prettily decorated with flags and drapery for the occasion, one side being devoted to the patients, the other to the visitors, of whom there were a good many present. Mr. de Barri Crawshay's two songs, "Fiona" and "The Holy City," were much enjoyed, and the patriotic sentiments expressed in "The Admiral's Broom," sung by Mr. Chappel Haverson, evoked special applause. Miss Sybil Noble fairly brought down the house, by her singing of "The Gay Tomtit" and "Mrs. 'Enry 'Awkins," and Miss Gethen recited one of her ever-popular and original stories of East-end life, "How Michael got took into Hospital." An amusing little farce, "The Silent Woman," given by Messrs. Andrews, Jones, and Thomas, opened the second part of the programme. The "German Band" (Messrs. Williams, Haverson, Rigby, and Worthington, in fearful and wonderful disguise), was received by the audience with significant but friendly hisses, which changed to much amusement when it opened proceedings with "Home, Sweet Home," and songs by Mr. Sydney Williams, Mr. L. A. Smith, and Miss E. Fisher, and violin solos by Miss Clara Fisher completed the programme. Refreshments were afterwards provided for the visitors.

### The Registration of Nurses.

With the object of ascertaining the wishes and views of the nursing bodies and training schools on the subject of the registration of nurses, a conference was held on Tuesday at the offices of the British Medical Association, 429, Strand, at which many of the institutions interested in nursing were represented. Mr. Ernest Hart, chairman of the Parliamentary Bills Committee of the British Medical Association, occupied the chair.

The Opinion of the Nurse Training Schools.

The Chairman: In opening this conference I need only state that it is called in virtue of the following resolution which was passed at the annual meeting of the British Medical Association in July last. The resolution was that "In the opinion of this meeting it is expedient that an Act of Parliament should, as soon as possible, be passed, providing for the registration and education of medical, surgical, and obstetric nurses; and the Council of this Association are therefore requested to consider this matter, and to take such measures as may seem to them advisable to obtain such legislation." That resolution came in due course before the Council, and was by the Council referred to the Parliamentary Bills Committee, of which I have the honour to be chairman. The Parliamentary Bills Committee considered that the first step towards the consideration of the matter was to ascertain what were the views and wishes of the nurses themselves and of the bodies for training nurses, and it was their desire that in the first instance we should have a conference with the representatives of the institutions interested in training nurses. A circular letter was sent round to them to that effect, and inasmuch as the resolution at the annual meeting was moved by Dr. Bedford Fenwick, I thought it desirable to ask him to state a little more precisely what was the exact meaning of his resolution, and what were the methods he had in view—in other words, what was his proposal, and what it was he wished to suggest for carrying it out. You have had put before you the memorandum which he wrote, and I will ask him to make a further statement of any kind he desires in further explanation of his views. There are a few letters, however, which, I think, should first of all be read. We have had communications from a certain number of bodies opposed to any registration, as follows: The St. Marylebone Infirmary Training School, the secretary of the Nightingale Fund, Westminster Training School, London Hospital, King's College Hospital, Charing Cross Hospital, Leeds Trained Nurses' Institute, and the Middlesex Hospital; and I have received the following communication, which summarises the objections of the bodies just mentioned. It reads thus: "At a meeting held at St. Thomas's Hospital, Albert Embankment, January 10th, at which were present Mr. J. G. Wainwright (treasurer of St. Thomas's Hospital), Mr. H. Bonham Carter, (secretary of the Nightingale Fund), Dr. Alchin (Westminster Hospital), Canon Troutbeck, Mr. H. Burdett, Mr. G. Q. Roberts (house governor and secretary London Hospital), Miss Lückes (matron, London Hospital), Miss Gordon (matron, St. Thomas's Hospital), Miss H. Gordon (matron, Charing Cross Hospital), Miss Monk (matron, King's College Hospital), Miss Pyne (matron, Westminster Hospital), Miss E. Vincent (matron, St. Marylebone Infirmary), and Miss Rosalind Paget (inspector, Queen's Jubilee Nurses), it was resolved, 'That this meeting of representatives of certain metropolitan nursing institutions and nurses' training schools having heard the resolution of the British Medical Association, which is thus cited, re-affirm the position hitherto taken up by them that a legal system for the registration of nurses is inexpedient in principle, injurious to the best interests of nurses, and of doubtful public benefit, and failing to recognise in the document circulated by the British Medical Association any reasons for altering their opinion, decline to enter on further discussion of the subject.'" That is signed by Mr. Jackson Hunt, who was in the chair. The other letters are individual communications to the same effect. I have here a longer letter from Mr. Bonham Carter giving arguments and reasons against the proposed registration, which I do not think I need read, but we will put it on record. I will now ask Dr. Bedford Fenwick to make any statement he wishes in support of the resolution he moved.

Dr. Bedford Fenwick: I did not expect to have the honour of addressing this conference, or of further explaining the views I have held and advocated during the past eight years, but I have very great pleasure in acceding to the Chairman's request to do so. With reference to the document you have just read from bodies opposed to the proposed registration of nurses, I think it is noticeable that, although certain statements are therein made, no proof is advanced in support of them. The question of the registration of nurses was brought forward a good many years ago, and it was taken up by a considerable section of persons interested in the training of nurses, and after considerable opposition—chiefly from the persons named by the chairman in the letter he has just read, and who everyone feels were actuated by the very best desire to promote the interests of the nursing profession—after, I repeat, great opposition, the matter was taken definitely before the Privy Council in the shape of a request for a Royal Charter for the Royal British Nurses' Association. After hearing the arguments their lordships decided to recommend Her Majesty to grant the charter, and in the preface to the preamble of it they practically stated that they considered the maintenance of a list of nurses, showing where each was trained and what training she had received, were points on which it was of advantage that the public should receive information. I think the effects of registration in other professions, the effects of informing the public who are and who are not qualified to follow the profession they profess to be able to follow, is of admitted public benefit, and that is all the registration of nurses comes to. It comes to, first of all, the publication of a list of nurses, showing when and where they have been trained and what definite training they have received, and then it gives to the body entrusted with all the details of registration the work of supervising the register and purging it, if necessary, of persons unworthy to remain on it. For six years the Royal British Nurses' Association has carried out such duties, and I think it is admitted that the work that association has done in this direction has been of a beneficial character. Nurses from all over the country have placed their names on its register. In at least one colony an Act has been passed making the registration of nurses a legal necessity for all who desire to practise as nurses there. Meanwhile, the association has gone on with its work under the powers granted by its charter in a quiet manner. The question of midwives having during the past year become very acute, and the feeling of medical men having gone against midwives being registered as properly qualified practitioners—it being suggested by them that they should be called midwifery nurses—the question arose whether all nurses should not be registered under some definite and clear rules. In these circumstances the reason becomes apparent for my having moved the resolution which was carried unanimously at the annual meeting of the British Medical Association in London last year. There is no doubt that the registration of nurses is bound, sooner or later, to be legalised by Parliament. It is felt that if an Act is to be passed at all, it should be on broad lines, so as to make it useful generally. If such an Act were passed, to whom was to be confided the power of registering and controlling the nursing profession? I suggest that a body should be formed for this purpose representative of all the interests involved. Of course, the scheme I have outlined in the memorandum I have circulated embodies only my own views, and must not be regarded as by any means of a final nature. I think it will be admitted, however, that my scheme provides for the representation of all the interests involved. First of all, there is the nurses' training schools; secondly, the medical profession; then the Privy Council, in accordance with precedent, should have their representatives; and, lastly, the registered nurses should have their elected representatives. The difficulty one has to face in dealing with this problem is the number of training schools that exist. In the case of the medical profession only hospitals with a hundred beds are allowed representatives, and I would limit the right of hospitals to be represented on the body in charge of the registration of nurses to those with a hundred beds. As there are about ninety-nine hospitals in the United Kingdom with a hundred beds that would give a very large and unworkable body, and so I propose that the full number should be constituted a governing body and delegate its active work and authority to a smaller body to be appointed by itself. I think this is all I need say, but if there are any further details or explanation I can give I shall be pleased to do so.

Institutions Represented.

The Chairman: I may say that the institutions represented here to-day are Royal National Pension Fund for Nurses, the Queen Victoria Jubilee Institute, Registered Nurses' Society, Royal British Nurses' Association, London Association of Nurses, St. John's House, Maternity Charity District Nurses, Plaistow, West Kent General Hospital, Nursing Sisters of St. John the Divine, Mildmay Nursing Home, White Cross Nursing Association, the Nursing Institute, Blackheath, and one or two others.

A Delegate: Then there is not a great training school represented?

The Chairman: No; they object.

Dr. Brown: May we take it they all object?

The Chairman: Those I have previously mentioned do. We have had no answer from Guy's.

Mrs. Bedford Fenwick: I should like to ask the chairman if, at the meeting at St. Thomas's, there were any representatives of the nurses themselves? It appears to me that there were secretaries and matrons, but so far as we hear there was no active nurse present. We have suffered much in the past from not getting the genuine opinion of the nurses from these institutions, owing to their not having been consulted. I think that is an important point. It appears to be rarely the custom to consult the nurses about matters which, after all, are for their own benefit. The time has gone by, I think all here will agree, when we women are to be entirely legislated for. We now require to be consulted about what we want, what we think right, and what we should like; and if we are not consulted by the heads of our institutions there is little doubt but that we shall express our opinions in another way. Now, as a representative to-day of the Registered Nurses' Society, I will touch for a moment on the industrial question, because it appears to me that it is now the industrial question in connection with nurses which is of such great importance. I have been doing for eighteen months a little honorary work in regard to the superintendence of the work of this Registered Nurses' Society. No nurse is admitted on to the roll of this society unless she has been trained for three years, and unless she has been registered by the board of the Royal British Nurses' Association. But what do I find? I find that these nurses, thoroughly competent and valuable servants of the public, go out and compete in the open market with probationers of one year's training from a variety of institutions, and, I regret to say, from the London Hospital, the Westminster Hospital, and, with little more training, from Guy's Hospital. As a rule, these institutions charge almost as much, or as much, as the thoroughly competent nurse would command. Now, both these systems are wrong. First, because the nurse is deprived of the regular training she should have to qualify her for a private nurse, and because she also takes the work away from the woman who has been conscientious enough to qualify herself thoroughly. Moreover, these institutions can afford to undersell the trained nurses. From an industrial point of view all this is bad. It is a thing medical men object to very much in their own profession. They do not care to meet unqualified men, and they certainly object to any underselling of their excellent work, and I do not believe that they would recognise it for a moment as a just or justifiable practice to be deprived of their full fees by the boards of those hospitals to which they belong. What is just for one sex is entirely just for the other, and it is from this point of view we shall have to consider the question of trained nurses. The nurses are a body which neither the public nor the medical profession can do without. Their work is of a very useful kind, and as good servants of the State they ought to have the consideration and protection of the State. The present system of voluntary registration is not a satisfactory arrangement altogether. We nurses ask for a minimum qualification. We want a definite curriculum of education, and definite standard of knowledge, and a definite examination, so that we shall know who is and who is not a qualified nurse. We are not content to be at the mercy of any body of hospital governors who choose to adjudicate on matters concerning us, because we find that many of them, although having the best intentions, are ignorant concerning what nursing is and what it should be. The *non possumus* attitude adopted by certain institutions with respect to the registration of nurses cannot last. They must be borne along by the great wave of progress, and, therefore, it is an attitude which appears to me to be a great mistake to adopt. The nurses themselves are asking for registration, and the public are beginning to understand the justness of it, and so it is bound to come. When it does come I hope that those gentlemen and ladies who hold representative positions, and who will be asked to organise the system of registration will recognise that direct representation must be given to the nurses themselves. That is the strongest point I wish to impress on this meeting, and I hope it will not be lost sight of by those in a position to deal with it.

A desultory discussion followed, in the course of which Mrs. Bedford Fenwick stated, in reply to Mr. Hemming, that the nurses would be willing to provide the money which State registration would entail. Dr. Woodcock, Manchester, urged that the registration of midwives was quite a distinct question from the registration of medical and surgical nurses, a view which was confirmed by Miss Wedgwood, who pointed out that all nurses could now be registered by the Royal British Nurses' Association if they chose to avail themselves of the privilege. Dr. Miller and others supported Dr. Woodcock's view, and a resolution was moved by Dr. Woodcock, and seconded by Dr. Ward Cousins, to the effect that the registration of midwives was an urgent public question, and should be kept distinct from that of general nurses; and that the question of general registration should be postponed until the question of the registration of midwives had been dealt with by Parliament. Sir Walter Foster, M.P., objected to this course on the ground that they had been asked to obtain the opinion of nursing societies in a resolution already adopted by the British Medical Association, and anything inconsistent with that would be contrary to the instructions given to the conference.

Miss Wilson: I wish to say a few words on the question raised affecting midwives, and let me say at once that I do not think the time is ripe for a Bill to be passed for the registration of nurses. We who work in connection with the Workhouse Nursing Association are aware of the need there is for training nurses. Powerful as is the industrial argument advanced by Mrs. Bedford Fenwick, the argument for the suffering patient must be a stronger one. Until the sick are properly cared for, whether able to pay or not able to pay, I do not think the question of legislation should be introduced. The annual reports of the *British Medical Journal* of the state of workhouse infirmaries in England and Ireland have been painful reading for a large number of us. The condition of things is almost incredible and most shameful, and while we speak of what may be done for paying patients we entirely forget the great need that exists in one of our great State departments where the sick are supposed to be cared for. It is impossible at present to get a proper supply of nurses for all our workhouse infirmaries. The pay is not large enough, and nurses are too few. Therefore, I think this question should be seriously considered. There is the large district nursing question, which also comes in. In regard to the question raised by Dr. Woodcock, I would say that I protest against the use of the term midwifery or obstetric nurse being applied to a woman who has charge of a woman in her confinement. Whether trained or untrained, she has charge of natural labour, and is not a nurse. She may be a nurse in part, but she is essentially a midwife, and the duties of the two are quite distinct. I think if old terms were adhered to the difficulties would not be so great as they appear to be.

Ultimately the Chairman put to the meeting a resolution to the effect that a legal system for the registration of nurses was inexpedient in principle, injurious to the best interests of nurses, and of doubtful public benefit. Only the representatives of nursing societies took part in the division, which gave six votes for the resolution and five against; so that the suggested State registration was negatived. The names of those voting will be found in another column.

Miss Kenealey: As a nurse I appeal to the medical profession here to help us to emerge from the state of quackery which, by the passing of the Medical Acts, they have freed themselves from. The increase in the number of quack nurses grows from year to year, and I think, in seeking to classify ourselves, we ought to be able to count on the medical profession. We nurses, I am afraid, have been too modest in the past, and we have therefore been treated on the principle that those who do not ask do not want. It appears to me that the time is now ripe for us to emerge from the modest violet stage, and go forward, and let the medical profession know what we want. If any woman prefers to be a quack, or to be known as an untrained nurse, let her stand to her guns, and refuse to put her name on a State register, but we who desire to be registered ought to have the opportunity afforded us. At the present time there are hundreds of unqualified women getting the same fees as trained nurses; but what is worse, there are hundreds of quack nurses going about the country representing themselves as hospital nurses who have never had a day in the hospital, and these women are teaching nursing without knowing anything about it. I should like to see some system of State registration which would prevent any woman from calling herself a nurse without the right to do so. It ought to be an honour to wear the nurse's uniform, but at the present time it is a disgrace to some extent. The present position of nurses is anomalous. It seems to me we are a little lower than governesses, and a little higher than parlour-maids. (Laughter.) I should like to endorse all that Mrs. Bedford Fenwick has said about the registration of nurses, and I should like to protest against the governors of hospitals coming forward and stating what nurses want. I think the nurses should be given the opportunity of themselves stating what they want, and that they should have the right of organising themselves into a definite profession. If the governor of a hospital—who is generally a layman—were to dictate to the medical profession what their professional position should be, it would soon be resented. We nurses equally object to any body of laymen saying what position we shall occupy.

Miss Stewart: As a representative of the Matrons' Council, which has connected with it about seventy-five matrons in London and the provinces, I think I may say that we are almost unanimously in favour of State registration, and for a great many reasons, one of which is the payment of nurses. I happen to be in a position to know a great deal about the payment of nurses, and such facts as I am going to state have come to my notice. I know one lady who has only had three months' training in London who gets two guineas a week like a certificated nurse. Such cases as that I have come across frequently. I think that is a great argument in favour of registration.

The conference closed with a vote of thanks to the chairman.

### Queen Victoria Jubilee Institute for Nurses.

Her Majesty has graciously approved of the following nurses being placed on the roll of Queen's Nurses:—

England—Superintendents: Annie Bradgate, Camberwell, S.W.; Charlotte Newman, Manchester; Ellen Macdonald, Tunbridge Wells. Nurses: Isabella Wilson, Emma M. Eldred, Mary Ingham, Elizabeth Shaw, Mary J. Love, and Jessie H. Matthews, London; Marian J. Coupland, Kingston; Helen Clayton, East Finchley; Beatrice Carr, Windsor; Mary W. Doe, Great Easton, Essex; Alice A. Headford, Easton, Essex; Ethel Grant and Florence Hargreaves, Peterborough; Edith J. C. Paice, Kettering; Kate L. Scudamore and Johanna M. C. Ludwig, Darlington; Gertrude F. Eastwood, York; Hannah Tyson, Rushden; Frances Chapman, Malvern; Edith M. Field, Louth; Harriette Green, Leamington; Adious Bradley, Brigg; Selina Walker, Pudsey; Alice H. Elkington, Bolton; Mary C. E. Bridges, Bath; Catharine E. Williams, Southampton; Emma E. Travis, Frances E. Merfin, Margaret J. Osborne, Mary J. Wallett, Hannah Grimes, Sarah Hughes, and Ada J. Austin, Birmingham; Ellen D'Arcy, Evelyn J. Brunt, Annie M. Breakall, Ruth Tyrrell, Mary Parsons, and Georgina A. Waters, Manchester.

England.—Rural Branch.—Nurse: Edith M. Rogers, Cornwall.

Wales.—Nurses: Elvira R. George, Katharine Roberts, Elizabeth E. Williams, and Elizabeth O. Heyward, Cardiff; Annie Roberts, Conway; Ellen Rowlands, Tregarth.

Ireland.—Nurses: Jeannette Peyton and Elizabeth M. Batty, Dublin.

Scotland.—Nurses: Joan B. Cameron, Lochaber; Cecile H. Gibert, Wick; Mary A. Lewis, Greenock; Ellinor Smith, Dundee; Mary Parker, Carluke; Barbara Milne, Barrhead.

### Our American Letter.

(Contributed.)

With us as elsewhere, Christmas and New Year have absorbed many thoughts and much time in advance, as well as at the immediate seasons. Both will be well passed ere this letter is printed, and English nurses, like their sisters in America, will no doubt have settled down to ordinary every-day life. It is not so much what each individually undertakes at Christmas that alters the common routine, but the all-pervading feeling that it is a great festival cannot be ignored.

Then there follows rebellion against sorrow and pain intruding themselves at such seasons, and hence we nurses, whose mission it is to fight, alongside the doctors, against disease and death, have our work cut out at Christmas. We have to do a little better and a little more than at other times, because on us largely depends our patients' view of the particular Christmas they spend with us. It is pleasant to feel that in spite of drawbacks many a sick man and woman will for ever remember gratefully "that Christmas Day when I was laid up," in hospital or home, as the case might be.

At the December meeting of the New York City Training School Alumnæ Association a delightful discourse on Swedish movements and massage by machinery was given by Dr. Carl Falcen, of the Zander Institute. A very pleasant gathering marked the distribution of diplomas to the graduates of St. Mary's Hospital, Brooklyn—a medal presented to each member of the class was engraved with the words "Be ye Faithful."

The appointment of superintendent of the West Penn. Hospital Training School, Pittsburg, has been conferred on Miss Katherine Emory, of the Elmira Hospital. Miss Mary Layton has taken the post of superintendent of the Protestant Infirmary, Lexington, temporarily.

A very large company assembled at the Metropolitan Trained Nurses' Club, New York, on the occasion of the annual social gathering. Many nurses appeared in uniform, a novel proceeding in the U.S.A. although so common in Europe. The appearance of the representatives of the various schools formed a pleasing tableau.

The new wards at the Woman's Hospital, Philadelphia, were inspected by many friends in December, when the exercises of the graduating class, of twenty-five nurses, took place. Several addresses were given, and a pleasant evening followed.

At the New York Cancer Hospital the post of clerk has been given to a nurse, and the superintendent, matron, and all other officers are nurses. It is announced that preference will be given to trained nurses applying for all vacancies at this institution.

Miss Leary resigned her position as superintendent of the Mount Sinai Training School last month, and a special reception was given in her honour, at which she was presented with a fine solitaire diamond ring as a token of the esteem in which she is held by the whole school. The subject of post graduate classes for nurses receives much attention just now, and are generally approved of.

The first six pupils who have completed two years' training at St. Vincent's Hospital, Norfolk, Va., received their diplomas recently. The school is under the superintendence of Miss Caroline Farnum, who was a graduate of Blackley Hospital, Philadelphia.

### Presentations.

On the eve of her marriage with Dr. Williams, M.A., Head Master of the County School, Abergele, North Wales, Nurse M. Woods was presented by Miss Murray (matron), Sister Bessie, and the staff of the Halifax Nurses' Home with many valuable tokens of their esteem and affection.

### The Winter Exhibitions.

Old Masters at the Royal Academy.

There are considerations besides love of art which induce busy people to visit picture exhibitions. Few things are more fatiguing than gazing at miles of pictures in a heated and exhausted atmosphere. This condition has to be counted with if the summer exhibition of pictures at the Royal Academy is visited. What a different aspect does the reposeful and refreshing winter exhibition of selected gems of art present to the busy and tired worker. No chattering crowd makes progress or a quiet study of a favourite picture well-nigh impossible, and "skying" is unnecessary with the adequate space available for the small and select company of pictures. The soft, warm tints of the paintings, which time has mellowed so harmoniously, form a soothing atmosphere.

The portraits now exhibited at the Academy are exceptionally interesting. The bygone company looking out from the canvases awaken memories and a retrospective interest which takes one far from present days. Robespierre, with his keen, satirical, but not unkindly face, portrayed by the celebrated French painter of children, Greuze; the Duke of Wellington, by our own prince of portrait painters, Sir Thomas Lawrence; Mrs. Herbert, by Romney; Madame de Pompadour, in wonderful apparel, painted by François Boucher; and Mrs. Siddons, by Sir Joshua Reynolds, all lend their aid to lead us back to past eras of history. Madame de Pompadour, though the painter has bestowed much skill in displaying the magnificent dress, garlanded with roses and enhanced by the sumptuous surroundings, reveals herself to us with a fresh-coloured intelligent face, with hair smoothly brushed from the temples, not unlike the Flemish in features and style. Robespierre is more kindly, the Iron Duke less commanding, than we might expect. Such portraits and others, such as, for instance, that of the brave Madame le Brun (painted by herself) who [supported herself and children, when left to battle with privation at an early age, are full of interest. The most beautiful of all is perhaps Romney's Mrs. Cook.

Many a familiar scene in history is recalled by one and another of the pictures. Paul Delaroche, the French painter, shows us "Cardinal Richelieu bringing back Cinque-Mars," and "Cardinal Mazarin's Last Illness." "The Library at Holland House," by Charles Leslie, and "A Conversation at Wanstead House," by William Hogarth, introduce us to the interiors of beautiul historical homes. Here, too, are pictures pleasantly familiar to many. Gainsborough's celebrated "Blue Boy" and Murillo's "St. Sebastian" are amongst these.

All the pictures I have mentioned are in the first three rooms. The fourth room is devoted to artists of earlier date, and the subjects are mainly religious. To those who have learnt to appreciate the meek and saintly faces portrayed by Fra Angelico, Filippo Leppi, and other artists of earlier periods, this last gallery will prove most interesting. Putting the cultivation of criticism in art aside, an appreciation of the earliest religious paintings and landscapes of a later period is the result of a special cultivation which those busy in other directions have seldom the time, taste, nor opportunity to pursue. Therefore we do not dwell here on the works of Gorgio, Gaddi, Lorenzetti, and many others.

Space, indeed, precludes the mention of numbers we would not intentionally pass by. There are examples from the brushes of our English painters, Constable, Etty, Morland, and Ward. The interesting painter of peasant life, Jean François Millet, and Rousseau amongst French painters also find a place in the present exhibition. The opportunity to make a better acquaintance with past masters of French art is welcome, as we in England are only now beginning to appreciate them.

Velasquez, the king of Spanish painters, Titian amongst Italian painters, and numerous others of the different national schools of painters are represented, and afford most delightful study. We cannot all be art critics, but such an exhibition must awaken more than the mere critical faculties. We may not be able to discuss the relative merits of a Claude and a Turner, but we are no less ready for that to admire the beautiful specimens of the work of both these and other artists.

Mrs. Ernest Hart's Pastels at the Dowdeswell Galleries, 160, New Bond Street.

Mrs. Ernest Hart has brought back with her about forty studies of "The Glories of the Sky and Sea from the Far East." The artist has chosen her medium wisely, for the vividness and brilliancy of pastels particularly lends itself to a close imitation of the real colours of nature. Long ago Mr. Ruskin remarked to us it was impossible to exaggerate the prismatic effects in the sky at sunset. In the present instance, Mrs. Ernest Hart has not exceeded truth in her sky effects. Glowing in all the thousand brilliant tints of the clearer atmosphere, her sketches from the Far East are truthful impressions of her subject. They are more than mere sketches and imitations; they have something beyond that about them—a sense of poetry which, in some instances, transforms them into poems.

And with perhaps one or two exceptions, all Mrs. Ernest Hart's drawings, here exhibited, are poetical; added to that, too, a certain daring of conception attracts us to them. No. 19, "Sunrise over the Snow Peaks of the Himalayas," is one of the best examples of her work. In the distance are "The Snows," some sixty miles off, whilst in the immediate foreground is a mist of purple mountains. Talking of foreground, it is there where Mrs. Ernest Hart shows want. The construction of her pictures and their colouring are good, but her foreground subjects undoubtedly show a weakness inconsistent with their general excellence. At these same galleries there is also a charming series of George Carline's paintings, "The Home of Our English Wild Flowers."

### Entertainments.

At the Victoria Infirmary, Glasgow, a very gay and happy Christmas was spent. On Christmas Eve carols were sung in all the wards from half-past six to half-past nine, the choir finishing in the lower corridor with "Hark, the Herald Angels Sing." On Christmas morning everyone had Christmas letters, and the children, of whom there were thirty-five, found large parcels of toys provided by Santa Claus. At noon a dinner of roast beef, chickens, and plum-pudding was served to the patients; the nurses and resident medical staff dined together at six p.m. A concert was afterwards given in one of the wards by the residents and nurses, bringing a happy day to a close. On December 27th a very large Christmas tree was placed in the hall, where all that could be moved were present to receive a useful gift from Father Christmas, who was personated by one of the staff. The tree, with decorations, toys, sweets, &c., was the gift of Mr. and Mrs. Crum, of Thornliebank; the useful gifts, which were contributed by the Victoria Needlework Guild and by the Guild of Kindness, gave a great deal of pleasure to the patients. Some of the women seemed quite lost in wonder at the beauty of the wards and of the tree. On January 4th a delightful tea party was given by the lady visitors, and the tables arranged in every ward were plentifully supplied with cakes, fruit, &c. The visitors afterwards entertained the patients with music, singing, recitations, &c.

The annual entertainment at the Metropolitan Hospital took place on the 9th inst., and the interior of the institution was gaily decorated and prettily illuminated on the occasion. There was a grand Christmas tree in the children's ward and a liberal supply of gifts for all the patients.

### Dr. Barnardo's Entertainment at the Albert Hall.

ON Saturday Dr. Barnardo gave the fourth of his Christmas entertainments at the Albert Hall in aid of the Young Helpers' League. The hall was crowded, and rarely has a better entertainment been witnessed within its walls than that furnished by the children from Dr. Barnardo's Homes.

This League is a society in which the children of the rich pay for the crippled, blind, and deaf children of the poor, who are to be found among the other waifs and strays provided for in Dr. Barnardo's Homes.

The entertainment consisted of 20 different items, and the band and orchestra were composed of the musical waifs, boys and girls.

We are often told that we English are an unmusical nation. Dr. Barnardo has evidently found the contrary, for his tightly-packed orchestra sang several songs with the sweetest of clear child-voices, and the playing of the band and of the ten pipers was a performance of which no full-grown musician need have been ashamed.

An acted song, "The King, the Queen, and the Blackbirds," was immensely appreciated, and we have rarely seen anything better done than the huge pie, out of which hopped four - and - twenty of the most comic-looking blackbirds imaginable. These birds hopped round the arena, upsetting the King in his counting-house and the Queen at her bread and honey, and then, in true story-book fashion, snapping off the maid's nose as she was hanging out a shirt to dry.

The cutlass drill by ninety boys dressed as sailors was a pretty performance, and the cheering was deafening as these small sailors marched away to the tune of "Rule, Britannia."

Ninety other little boys dressed as soldiers went through battalion movements (receiving command by bugle) with the greatest precision, even firing their small rifles, and certainly we as a nation owe the Doctor thanks for implanting into these children—children of a class so low that they belong to no class at all—a love for "playing" at soldiers and sailors. Of the healthfulness of the drilling and the gymnastic performances that both girls and boys gave specimens of there can be no question, and the straight little bodies, rosy cheeks, and the clean, well-kept appearance of the children, almost prevented one realising that these were the children who had actually begun life in rags and a gutter.

The entertainment ended with several lime-light pictures showing the various industries in which the waifs are engaged. The last picture, the Jubilee portrait of the Queen, was received with deafening applause. Dr. Barnardo evidently brings up his large family of nearly five thousand as loyal and enthusiastic subjects of the Queen.

We are told that from forty to sixty "unwanted" children are added to the Homes every week, and that during the twenty-nine years that these Homes have been in existence more than twenty-eight thousand children have been saved from a life of misery and crime, and started in a new life as self-respecting Englishmen and Englishwomen.

### Lectures to Nurses.

### THE CHELSEA HOSPITAL FOR WOMEN.

THE following course of lectures were given during the third session of 1895:—

AUGUST—By ARTHUR GILES, M.D.—Lecture I. Anatomical Principles: The Skeleton; II. Skeleton (continued): Joints, Muscles, and Tendons; III. The Heart and Lungs; IV. The Abdominal Viscera; V. The Nervous System; VI. The Female Pelvis and Pelvic Organs.

SEPTEMBER—By T. W. EDEN, M.D.—Lecture I. Physiology of the Blood and Circulation; II. Respiration; III. Digestion; IV. Excretion: Kidney and Skin; V. Physiology of the Nervous System; VI. The Nervous System: Regulation of the Body Temperature.

The following is the course of lectures for the first session of 1896:—

Lectures on Nursing. — By J. INGLIS PARSONS, M.D. —January 15th.—Lecture I. The Principles and Practice of Antiseptics: Aims, Solutions, Cleansing Hands, &c., Dressing Wounds. January 22nd.—II. Gynæcological Nursing in General: Douching, Plugging Vagina, Enemata, the Catheter. January 29th.—III. Nursing of Cases of Minor Gynæcological Operations after Treatment: Perinæorrhaphy, Curetting, &c. By WILLIAM FENTON, M.D.—IV. The Preparation for Abdominal Section: (a) Preparation of the Patient, (b) Preparation of the Room, (c) Preparation of Instruments, Sponges, Solutions, &c.; V. The After-Treatment of Abdominal Section: Ovariotomy, Hysterectomy, Kidney Operations, &c.

### Everybody's Opinion.

[Correspondence on all subjects is invited, but we cannot in any way be responsible for the opinions expressed by our correspondents. No communications can be entertained if the name and address of the correspondent is not given, or unless one side of the paper only be written on.]

### THE PRIVATE NURSE.

"NURSE KIRKMAN" writes from Paris: May a private nurse of six and a-half years' standing be allowed to give her views on the subject of a private nurse? My experience is that this said nurse should, if possible, be a gentlewoman, well educated and refined, most certainly not one of the class of "upper housemaids." Has "Sister Grace" worked much in the capacity of private nurse? And if so, can she be aware that they are not called in only to have nice little ways of waiting on their patients? Surely a nurse obtains this by instinct, if not during her hospital training; for often the nurse is the only person to whom the invalid can rely upon for companionship, &c. Besides her nursing, how many hours are spent by her reading to her patient in the long weary days of convalescence. Would it be possible to obtain this from an upper servant in the same way an educated nurse could do it? Also most of the correspondence is carried on through the nurse. There cannot be too much care taken in selecting suitable nurses for private patients. I may say, in conclusion, that in no other sphere of life is a congenial spirit more needed.

### THE TITLE OF NURSE.

SISTER KATHERINE TWINING writes: I am very glad that you are urging upon your readers the necessity of having two grades of nurses. The word "nurse" is too homely and comforting to be parted with in a hurry, and we cannot entirely give it up to the modern hospital nurse. It would never do to deprive a man of his right to be called "soldier" who was fighting for his country, on the grounds of his having enlisted for short service.

[We agree with Miss Twining that every man in the army has a right to the name of "soldier," whether he is a recruit or a veteran, for in the first case he has enlisted with the sole idea of becoming a thorough soldier. But there can be no logical comparison between the young soldier who is learning his profession and the woman who, after a few months' instruction, intends to leave her corps and adopt the uniform, title, and position of a skilled "nurse." It is the very fact of its being "homely and comforting" which makes this name so misleading and dangerous. We look forward to a day when the generosity of the public will enable Miss Twining to decline all probationers who will not consent to remaining with her for two years' continuous training. Her noble work at Plaistow deserves support and encouragement, and when this is given in an adequate degree her supply of district nurses will probably make her independent of the service now only obtainable for the destitute poor by the co-operation of short time probationers with her trained staff nurses.—ED. *T. H.*]

### Royal British Nurses' Association.

The following resolution was carried unanimously at the meeting of the general council on the 10th inst.: "That the Executive Committee sanction the recommendation of the Royal National Pension Fund to members, both by means of its literature, through the official organ of the Association, and by personal influence, the committee being convinced that this course would assist the members in the exercise of thrift, until the Association is in a position to form a fund of its own for their assistance in old age."

A special general meeting of members of the Corporation will be held, by command of H.R.H. the President, at 20, Hanover Square, W., on Tuesday, January 28th, at four p.m. The subject of Dr. Colman's third lecture on the "Nursing of Nervous Diseases," on Thursday next, at three p.m., at the offices of the Association, is "Chorea, Delirium, and Hysteria."—Alice Ravenhill, secretary to the corporation.

### Notes and Queries.

The contents of the Editor's Letter-box have now reached such unwieldy proportions that it has become necessary to establish a hard and fast rule regarding Answers to Correspondents. In future, all questions requiring replies will continue to be answered in this column without any fee. If an answer is required by letter, a fee of half-a-crown must be enclosed with the note containing the enquiry. We are always pleased to help our numerous correspondents to the fullest extent, and we can trust them to sympathise in the overwhelming amount of writing which makes the new rules a necessity. Every communication must be accompanied by the writer's name and address, otherwise it will receive no attention.

**Queries.**

(75) *Co-operation.*—Can you supply me with the address of The Nurses' Co-operation, London?—*Nurse Lamond.*

(76) *Lady Roberts.*—I have written to the addresses given me, but learn that they apply to the Indian Nursing Service, and not to Lady Roberts' nurses. Can you help me further?—*Sister Alice.*

(77) *Specialist.*—I should feel obliged if you could inform me of a specialist for women's diseases in Liverpool. The patient is in moderate circumstances?—*L.R.C.P.*

(78) *Advice Gratis.*—Am I entitled, as a trained nurse, to medical attendance from any doctor gratis?—*H. S.*

(79) *Anæsthetics.*—Would a doctor one nursed for make any charge for administering ether for extraction of teeth?—*H.*

(80) *Cottage Nurses.*—What is the address of the association at Plaistow which supplies these nurses?—*M. K.*

(81) *Training.*—Where can an educated woman get training in workhouse infirmary nursing?—*M. K.*

(82) *Army and Navy Nursing.*—How can I get into either the Naval or Military Hospitals?—*Trained Nurse.*

(83) *Midwife.*—What is the best midwifery training school?—*A. A. M.*

(84) *"The Hospital" Convalescent Bed.*—How can admission to The Hospital Convalescent Bed be obtained?—*C. E. B.*

(85) *Stamp Collector.*—Can you advise me as to selling used English and foreign stamps for charitable purposes?—*J. E. S.*

(86) *Massage.*—Where can I obtain practical instruction in massage?—*Outsider.*

**Answers.**

(75) *Co-operation (Nurse Lamond).*—The address of the association is 8, New Cavendish Street. You will find the advertisement in our columns.

(76) *Lady Roberts (Sister Alice).*—You had better read what Lady Roberts stated in The Hospital Nursing Mirror of July 1st, 1893, p. cxxxvi. You will then find that she does not desire to receive applications from nurses. We regret that you have been wrongly informed.

(77) *Specialist (L.R.C.P.).*—We cannot name a practitioner, but no doubt the names of specialists could be ascertained on inquiry at the special department at one of the hospitals.

(78) *Advice Gratis (H. S.).*—Free medical attendance is not "the right" of anyone who can afford to pay for it. Whilst working in hospitals or kindred institutions nurses always receive free treatment, but afterwards we do not imagine they have any more claim than other independent women. At the same time, we constantly hear of the liberality with which the medical profession treats nurses who have at any time worked for them.

(79) *Anæsthetics (H.).*—Your best plan would be to ask the doctor what his usual fee is for giving ether. It is always better to do this, and it shows that you do not demand as a right what he may grant as a favour.

(80) *Cottage Nurses (M. K.).*—Address Sister Katherine, St. Mary's Maternity Home, Plaistow, E.

(81) *Training (K.).*—Write and ask matrons at St. Pancras Infirmary, Highgate, and St. Marylebone Infirmary, Notting Hill.

(82) *Army and Navy Nursing (Trained Nurse).*—Apply to the War Office or Admiralty for particulars.

(83) *Midwife (A. A. M.).*—Get "How to Become a Nurse" from the Scientific Press, 428, Strand; you will then be able to make a selection, and will find all the information you require.

(84) *"The Hospital" Convalescent Bed (C. E. B.).*—Apply by letter to the Hon. Secretaries, 428, Strand. All cases are considered on their merits.

(85) *Stamp Collector (J. E. S.).*—Theodore Buhl and Co., 11, Queen Victoria Street, E.C., purchase old stamps; but is it not rather a waste of time and your friend's patience to collect for this purpose, seeing the absurd prices given? Your 59,000 would only fetch two or three shillings. We believe that it is a fallacy that any institution receives a patient in return for used postage stamps.

(86) *Massage (Outsider).*—Write to the Hon. Secretary, Society of Trained Masseuses, 12, Buckingham Street, Strand.

### For Reading to the Sick.

ENDURANCE.

**Motto.**

"Stand firm like a rock against which the waves batter, yet it stands unmoved till they fall to rest at last."—*Marcus Aurelius.*

**Verses.**

In the world's broad field of battle,
In the bivouac of life,
Be not like dumb driven cattle!
Be a hero in the strife. —*Longfellow.*

Lord, who has suffered all for me,
My peace and pardon to procure,
The lighter cross I bear for Thee
Help me with patience to endure. —*Cowper.*

Mortal! thou standest on a point of time,
With an eternity on either hand;
Thou hast one duty above all sublime,
Where thou art placed, serenely there to stand.

To stand! undaunted by the threatening death,
Or harder circumstance of living doom,
Nor less untempted by the odorous breath
Of Hope, that rises even from the tomb.
—*Lord Houghton.*

**Reading.**

"We may long and pray that this cup pass from us . . . but when it is come, then thou knowest that this, and no other, is best for thee; else His wisdom had not chosen it, nor His love given it to thee to drink. It is a very dangerous deceit of impatience to think we could be patient under any other than what God gives us. Could we be purified or saved in any other way? Would He who saith 'He doth not afflict willingly, nor grieve the children of men,' have chosen this for thee? Be not, then, impatient with thyself, even that thou feelest impatient or hateful in thine own eyes; but, mourning for thy past sins, commit thy present and future, thy time and thine eternity, into His merciful hands who out of love made thee and redeemed thee, and now of love would re-make thee to His praise and thy bliss in Him."—*E. B. Pusey.*

"When sorrow comes to you, or to those you love, do not shrink from it as from some cruel torturer, but welcome in it an angel to bring you near to God. Do not fear—do not fear. 'This light affliction, which is but for a moment, worketh for us as a far more exceeding and eternal weight of glory.' Our God is enough for every one of us; and when we pass behind the cloud it is that we may see His face, and our joy may be full."—*Bishop Thorold.*

### Appointments.

Hospital and Dispensary, Newark-on-Trent.—Miss Finlay has been appointed Lady Superintendent of this hospital. She received her training at the Nurses' Home and Training School in connection with the Royal Hospital, Belfast.

Sussex County Hospital.—Miss Katharine Scott has been appointed Matron of this hospital. Miss Scott was trained at the Sussex County Hospital, afterwards holding the posts of staff nurse and ward sister. She has since been matron of the cottage hospital in connection with the Western Dispensary at Brighton. We wish Miss Scott every success in her new sphere of work.

### Minor Appointments.

Huddersfield Infirmary.—Miss M. E. Massey, who was trained at the London Hospital, and was afterwards assistant nurse at the Poplar Hospital for Accidents, has been appointed Charge Nurse of the male medical ward and Superintendent Nurse of the operating theatre at the Huddersfield Infirmary.

